# Public and commercial more of the same? The characteristics of the opening monologues of public and commercial channels during the COVID-19 pandemic

**DOI:** 10.1371/journal.pone.0293172

**Published:** 2024-02-14

**Authors:** Tal Laor

**Affiliations:** School of Communications, Ariel University, Ariel, Israel; Reichman University, ISRAEL

## Abstract

The novel coronavirus, COVID-19, first identified in Wuhan, China, in December 2019, rapidly spread across the globe and resulted in significant public concern. In response, numerous countries have implemented guidelines aimed at promoting social distancing, limiting movement and gatherings, instituting lockdowns and curfews, and closing institutions. As a result, the media, including news releases, has become a primary source of information for many individuals (Laor & Lissitsa, 2022). The present study seeks to investigate differences between major television programs featured on public and commercial channels in Israel, utilizing the lens of framing theory. Specifically, twelve monologues broadcast simultaneously on both channels were analyzed to compare differences in content, topics, format, and accompanying visuals. The study’s results indicate that both commercial and public channels exhibited similar behavior in shaping public opinion during the crisis period. Specifically, both channels displayed criticism of the government’s conduct and utilized negative framing techniques while neglecting to provide space for popular voices. Nevertheless, certain distinctions between the commercial and public broadcasting spheres still persist, particularly in the realm of visual presentation, as some long-standing elements that typify each channel have endured over time.

## Introduction

Various broadcasting models exist around the world, with the most prominent being public broadcasting and commercial broadcasting. Public broadcasting, which initially dominated the mass media landscape, aims to provide comprehensive representation of all subgroups within a country while allowing for the expression of a diverse range of opinions and cultures [1]. Consequently, public news programming often incorporates elements that may foster civic thinking, political knowledge, democratic values, and public capital, as public entities are not subjected to commercial pressures and receive state funding [2]. In contrast, commercial broadcasting entities rely on self-financing and therefore prioritize maximizing profits, often through sensationalized reporting and appealing to the widest possible audience to engender a sense of closeness and identification among consumers while preserving the existing social order [3].

The present study is grounded in the theoretical framework of framing theory, which posits that media coverage of issues can be framed positively or negatively while providing new perspectives and connections to consumers, potentially reinforcing pre-existing beliefs and empowering them indirectly [4]. Framing theory is particularly well suited for comprehending the impact of news media content in relation to topics that are subject to multiple interpretations [5].

During times of crisis, the media can play a vital role in either calming or exacerbating panic and thus has the potential to prevent the escalation or worsening of a crisis [6]. The COVID-19 pandemic has resulted in a significant increase in the use of traditional media, with many individuals, including younger users, returning to these sources for information. Furthermore, privious research suggests that television has been a more frequently utilized source of information than social media platforms [7]. Arguably, the COVID-19 pandemic has been the largest crisis of the 21st century to date, prompting the implementation of unusual government responses. Additionally, the modern public in Western countries has become more global, critical, individualistic, educated, and capable than in previous eras. As a result, the pandemic has forced a shift away from globalization, freedom of movement, and individuality.

During the COVID-19 pandemic, the Israeli government was the first to administer COVID-19 vaccines, and Israel has one of the highest vaccination rates in the world [8]. However, despite this success, numerous demonstrations have occurred in Israel in protest against government officials and the health care system [9]. These protests have targeted social distancing restrictions, the vaccination policy, and the COVID-19 certification process [10]. Israel provides an ideal case to study the mainstream media’s coverage of COVID-19 due to its successful vaccination campaign, technological innovation, implementation of public health measures and concentrated media landscape.

The significance of this study lies in the analysis and understanding of how the media frames a crisis, particularly on traditional communication channels such as television, which become even more dominant during such periods. To achieve this objective, a comparative analysis of 12 monologues broadcast simultaneously on public and commercial channels was conducted, analyzing the content, topics, format, and visuals utilized in the framing of the crisis. The research aims to contribute to the existing body of knowledge on media and crisis communication, particularly in the context of the differences between public and commercial broadcasting. Furthermore, it provides valuable insights into the influence of media ownership and organizational structures on crisis framing.

## Literature review

### Differences between public and commercial channels

Public radio broadcasting differs from commercial radio broadcasting in both form and substance. Public radio broadcasting is a non-profit model that is publicly funded and perceived as a public good. The primary objective of public radio broadcasting is to provide educational, entertainment, and cultural content, as well as to serve as a source of important information for consumers and a platform for the expression of diverse opinions and topics of public interest [11].

The social significance of public broadcasting stems from its function as a public sphere where individuals can actively participate in community life, affect decision-making processes, and engage in civic discussions [11–13]. Public broadcasting’s responsibilities include providing information beneficial to society, educating the public on cultural matters, promoting local innovation and content, and catering to diverse audience demographics [11, 13–15].

In Israel, public broadcasting has been the dominant mass media since the establishment of the state, with the aim of providing adequate representation of all subgroups in the country and giving expression to a variety of opinions and cultures. This policy persisted in Israel until the late 1980s [1].

In the 1990s, Israel saw the development of commercial broadcasting, which emphasized the values of competitiveness and private ownership of the media [2]. This marked a transition from an ideological-service orientation to a business-profit orientation [16]. The introduction of the first commercial television channel, Channel 2, at the end of 1993 had both short-term and long-term effects. In the short term, the entry of the commercial channel had significant economic effects. However, in the long term, consumers of broadcast content were influenced by norms of competitiveness, compensation, and pricing that were expressed socially [16].

Despite the emergence of commercial channels, the public channels in Israel continued to prioritize cultural programs and reliable information, in contrast to the entertainment or commercial programs broadcast on commercial channels [1]. However, the profitability of commercial channels through advertising revenue put pressure on the government to allow the opening of additional commercial television channels [2, 17].

In the context of the development of commercial broadcasting in Israel, a process of "individual disempowerment" was created to try to reach a diverse population. Despite the development of niche channels, most consumption is still concentrated in commercial channels, leading to a distorted market of opinions and reduced media ideological diversity [2, 18]. The dominance of commercial interests also harms the competitiveness of other channels, creating a further reduction in media diversity [19]. In this context, public broadcasting channels struggle to compete with commercial channels for ratings and sometimes target programs that emphasize their "scarcity of resources" [1].

Not all news media produce similar effects. In Western Europe, individuals’ civic attitudes may vary depending on their preference for either public or commercial news [20].

In a study conducted by Jacobs et al. (2016) that examined the attitude of viewers toward immigration based on their preference for public or commercial news, evidence was found that people who prefer public news are more positive about the issue of immigration than viewers of commercial news [20]. The study also found that commercial broadcasters address television viewers as customers or consumers, while public television news broadcasters address viewers mainly as citizens [2, 20]. Moreover, TV news is predominantly negative in tone, focusing on negative outcomes and common problems. However, in public news, the frequency of negativity is lower and there is more of a positive tone compared to commercial news, although positive news is still relatively infrequent [2, 20].

### Framing theory

Framing, a crucial practice in news coverage, involves selective topic choice and a subset of information that is presented to audiences, with some information remaining excluded from coverage [21, 22]. This practice limits the range of possible interpretations available to consumers and directs their attention toward what the media considers to be "important details" [23]. In traditional media, framing crises often incorporates negative words such as "panic" and "fear" to attract consumer attention, which, in turn, can affect their level of anxiety [24]. By choosing spokespersons and emphasizing risks and uncertainties, media outlets can either increase or decrease the level of panic in society. For instance, media outlets can interview experts who suggest various solutions or explain how to stop a contagion, or they can focus on speculations, blame-throwing, and risks [24].

Television news utilizes different framing practices that incorporate visual content alongside verbal content, as visual images tend to be more memorable than verbal elements. Additionally, moving images have a stronger emotional impact than still images. News content is often presented through stories that combine visual and verbal information, with visual information often having a greater impact than verbal information. Furthermore, negative information is more prominent and memorable than positive information, and counterarguments are more persuasive than professional arguments [5].

During crisis situations, decision-makers face the challenge of making critical decisions while under pressure and uncertainty, as stress and uncertainty can generate a strong public demand for information [25]. Mass media plays a crucial role in conveying information to the public, as it is responsible for creating order out of facts and stories [26]. Moreover, media coverage influences the public’s understanding of the crisis and its implications, which highlights the power of media not only in conveying facts but also in shaping the public’s perception of the crisis [21, 27].

In crisis situations, the media’s narratives and frames have a significant impact on the public’s perception of the crisis and the government’s policy, which, in turn, can affect consumer willingness to comply with government instructions [27]. As such, the media plays a critical role in either fueling criticism of government actions or mobilizing the public to support the government by providing a platform for the government’s voice [28].

During crisis events, journalism can be categorized into two types of coverage that can impact the nature of crisis reporting and journalistic practices: an inside-the-media perspective, where the crisis is described as a "surprise crisis" characterized by uncertainty and the unpreparedness of journalists. In such situations, journalists may struggle to remain objective and balanced due to their lack of preparation, leading to an adoption of the dominant government voice [27]. This is supported by the common use of official sources in crisis reporting [23]. On the other hand, an outside-the-media perspective describes the crisis from the viewpoint of external actors who provide clear and well-known stories to journalists. In such cases, it is easier for journalists to maintain balanced reporting [25].

In the contemporary era of social media, journalists have become exceedingly active on these platforms, leading to a blurring of the distinction between news producers and consumers. As a consequence, consumers are now exposed to not only the personal lives of journalists but also their personal opinions on a diverse range of issues. Consequently, in certain instances, the notion of "journalistic objectivity" has become obsolete and is no longer deemed relevant [29].

### Media and trust in government

Trust in government refers to citizens’ confidence in the ability of government action to result in outcomes that align with citizens’ expectations and preferences [30]. The decline in trust in government can be attributed to a range of cultural, political, economic, and other factors [31, 32].

However, the impact of media on trust in government is a topic of ongoing debate in the research literature. The increasing superficiality of mass media content has been identified as a major contributor to the loss of public trust, as media consumers have become accustomed to viewing politics through an entertainment-focused lens. In an effort to attract media consumers and increase their exposure and ratings, news coverage places greater emphasis on sensational stories, personal intrigue, scandals, and emotional discourse rather than on "pure" politics [33].

According to malaise media theory, the media’s focus on entertainment and sensationalism leads to political alienation and cynicism among consumers, ultimately undermining trust in government [31, 34].

This is exemplified by the situation in Israel during the COVID-19 pandemic, where traditional media was the primary source of information related to the pandemic. Research suggests that these media channels work together to shape reality for consumers, negatively impacting trust in government [35].

On the other hand, virtuous circle theory posits that the availability of a large amount of information through mass media can lead to increased citizen engagement in politics and participation in public discourse, potentially resulting in higher voter turnout. This theory suggests that a higher level of political involvement leads to greater trust in democracy, political processes, and government [36]. According to this theory, there is a cyclical effect where politically active individuals consume more political content and information, leading to greater trust in government and further political involvement and consumption of media content [36].

In conclusion, media organizations play a crucial role in the dissemination of information during crises and emergencies, particularly in the case of public health crises where the public relies on media for accurate and timely information. However, the media holds significant power in deciding what information is conveyed and how it is presented, and the COVID-19 pandemic serves as an intriguing case study for exploring the differences between commercial and public communication channels.

### COVID-19 coverage

During times of crisis, the general public actively seeks information, often relying on the media as their primary source. Consequently, media consumption significantly increases, emphasizing the importance of understanding the nature of media coverage. Studies investigating media consumption in relation to the coronavirus have established a correlation between consuming commercial media and heightened levels of anxiety and trauma indicators [37, 38]. Media outlets across fifty nations globally have disseminated content related to the coronavirus, covering topics such as hygiene practices, preventive measures, social distancing guidelines, lockdown protocols, vaccination campaigns, fatality rates, infection statistics, and other pertinent information [38, 39].

Moreover, the media often faces criticism for its tendency toward excessive, inaccurate, or potentially deceptive and biased coverage [40]. Various research studies conducted in the United States have successfully identified distinct framing elements adopted by the media when reporting on the coronavirus. Noteworthy frames include the economic frame, which presents the coronavirus within the context of its economic repercussions on individuals, societal groups, and the nation as a whole. Additionally, the human interest frame emphasizes personal stories and narratives, while the conflict frame portrays the pandemic as a metaphorical war. The blame frame attributes responsibility to various factors, whereas the religious and ethical frame explores the influence of the coronavirus on human behavior, moral values, and societal norms [41, 42]. Interestingly, media coverage during the initial wave of the coronavirus predominantly focused on elderly and vulnerable populations, presenting their isolation from society as a moral imperative [43, 44].

The Covid-19 pandemic witnessed a notable upswing in social media utilization, as evidenced by scholarly research [45]. During this period, numerous studies have delved into the intricate dynamics of information dissemination within the media landscape, with a particular focus on the realm of social media. Notably, social media platforms emerged as primary conduits for information-seeking endeavors, communication, and entertainment, especially in the context of social isolation. Furthermore, social media has come to embody a reflection of societal realities and challenges, shedding light on prevailing circumstances [40]. However, it is crucial to recognize that social media’s ubiquity also renders it susceptible to a range of potential pitfalls, including the proliferation of offensive content, dissemination of false information, and the propagation of conspiracy theories [45, 46].

## Research question

How does traditional media frame and shape a crisis in relation to its specific characteristics?

## Research hypotheses

Hypothesis #1: We hypothesize that there will be differences in content between the commercial and public channels.

Based on the literature review, which states that in public news, the frequency of negativity is lower and there is more of a positive tone compared to commercial news [20, 47], we assume that the commercial channel will use a higher frequency of negative and emotionally charged framing compared to the public channel, as reflected in its choice of terminology, tone, images, and video.

Hypothesis #2: We hypothesize that there will be differences in format/structure between commercial and public channels.

Based on the literature review, which states that negative information is more prominent and memorable than positive information and that counterarguments are more persuasive than professional arguments [5], we assume that the commercial channel will utilize a higher frequency of confrontational, counter argumentative, and negative messaging, while the public channel will prioritize neutrality, objectivity, professionally presented arguments, and in-depth investigations with a focus on democratic messaging.

## Methodology

This study employed a semiotic content analysis research method to compare and contrast the framing, text, and expression in the opening monologues of news broadcasts covering the spread of the COVID-19 pandemic between a commercial channel (Channel 12) and a public channel (Channel 11). For the purpose of the research, 12 segments were sampled from the leading commercial channel in Israel (Channel 12) and the sole public media channel in Israel (Channel 11) between June 19, 2020, and February 26, 2021, covering the COVID-19 pandemic and various lockdowns in Israel. In the selected segments, the content, text, titles, visual signs, and visibility were analyzed.

Each monologue was broadcast on the main weekly program (weekend edition) and presented by the host of the program, an experienced and senior journalist with high prestige and influence: Yaron Dekel on the public channel and Danny Kushmaro on the commercial channel. The coverage and commentary focused primarily on the situation in Israel, with references to the rest of the world and other countries regarding the crisis.

This study employed a semiotic content analysis research method to compare and contrast the framing, text, and expression in the opening monologues of news broadcasts covering the spread of the COVID-19 pandemic between a commercial channel (Channel 12) and a public channel (Channel 11). The research question seeks to understand how media framing shapes public perceptions of the COVID-19 pandemic [48]. Semiotic content analysis is a qualitative research method that involves analyzing signs, symbols, and texts to identify underlying meanings [49]. This approach is particularly useful for analyzing media content and has been used to examine a range of media phenomena, including news coverage, advertising, and political communication [50].

To achieve this objective, 12 segments were purposively sampled from the leading commercial channel in Israel (Channel 12) and the sole public media channel in Israel (Channel 11) between June 19, 2020, and February 26, 2021, covering the COVID-19 pandemic and various lockdowns in Israel. The selection of these segments aimed to capture a comprehensive range of news coverage from both channels and enable the researchers to compare and contrast the content of the monologues.

To conduct the analysis, the researchers employed a semiotic content analysis approach that involved analyzing the content, text, titles, visual signs, and visibility of the selected segments. Each monologue was broadcast on the main weekly program (weekend edition) and presented by the host of the program, an experienced and senior journalist with high prestige and influence: Yaron Dekel on the public channel and Danny Kushmaro on the commercial channel. The coverage and commentary focused primarily on the situation in Israel, with references to the rest of the world and other countries regarding the crisis. The data analysis was guided by the research question, and the researchers identified relevant themes and patterns in the data to draw comparisons between the two channels. The quality of the data collected, as well as the rigor of the analysis process, will determine the accuracy and reliability of the study results.

The approach of employing semiotic content analysis has the potential to provide insightful findings that can inform media practices and policies.

## Findings

Red cities: use of words and symbols from the world of COVID-19.

The use of symbols has been identified as a framing strategy in monologues, as they serve as meaningful and powerful representations that influence public opinion and "shape reality" [51, 52]. The current reality of the COVID-19 pandemic is reflected in the frequent use of words that have become prevalent in public discourse, such as "masks," "lockdowns," "carriers," "the COVID-19 project manager," "reliefs," and "red cities." The language of the monologues adapts to the current reality, and there is not a single monologue on the commercial channel that does not mention a phrase related to the COVID-19 reality at least once. On July 3, 2020, the term "corona" was used ten times, and on July 24, 2020, it was used fifteen times. The monologues also include symbolic icons related to the COVID-19 reality, such as a syringe and a virus, and images and videos that visually reflect the concepts of the COVID-19 virus.

The use of COVID-19 words and concepts is also evident in the segments of the public channel. On June 19, 2020, words such as "mask," "corona cabinet," "regulations," "virus," and "tests" were used. On July 22, 2020, nine words from the COVID-19 domain were used, including "lockdowns," "verified patients," "morbidity," and "red cities." Even after the arrival of vaccines, the language of the COVID-19 virus remained prevalent, and starting from January 22, 2021, to February 26, 2021, "vaccinates," "vaccines," and "Pfizer" were added to the COVID-19 vocabulary.

## Use of visuals

The use of visuals in communication is essential in creating a strong emotional impact on audiences. While verbal information can influence viewers, visual information is more influential due to the ease with which it is encoded and retained in the human brain. Visual memory is more accessible and more readily available than verbal memory, making it a more powerful tool in conveying meaning. A larger image tends to be perceived as more important by the audience, and the emotional framing it creates can have a significant impact [5].

In television news, visual images are remembered more than linguistic information and produce a stronger emotional impact [5]. The use of visuals, including pictures and videos, significantly differs between commercial and public channels. Commercial channels tend to emphasize the use of visuals, including pictures and videos, related to current events such as the pandemic or political situations. This emphasis on visuals aims to increase the viewer’s acceptance of the text and data presented and to convince them of the truthfulness of the message. Visuals that match the topic described strengthen the message, such as showing a certain politician in the background or pictures from hospitals and people wearing masks for news related to the pandemic.

On commercial channels, monologues often contain a multitude of images and videos that emphasize the importance of visuals in conveying a message. In contrast, on a public channel, monologues are short and concise, with minimal use of visuals. Initially, the monologues on the public channel were delivered with the host sitting in a chair. However, as the COVID-19 pandemic progressed, the host began to stand and present short segments, often accompanied by visuals such as images, videos, and graphs. Nevertheless, the use of visuals on the public channel was relatively minimal compared to the commercial channel. The use of titles throughout the monologues was also noticeably different in the two channels. The titles of the monologues on commercial channels often use terms related to the pandemic, with a negative connotation, which corresponds to the general mood of the monologues based on criticism and the use of negative words. On public channels, monologues are short and concise, with no title.

Commercial channels aim to combine effectiveness and efficiency to increase their audience [17]. Therefore, the news of commercial channels contains characteristics that emphasize sensational news features, such as news containing conflicts, as big headlines and graphs have the power to create a sense of significance and importance in news stories [20]. The use of large numbers and percentages, as well as graphs, is also prevalent in commercial channels. These elements help to illustrate complicated information in a flowing and simple way for viewers. The use of titles creates a significant framing for monologues that represent certain aspects of a perceived reality and make them more prominent in a way that emphasizes a causal interpretation or a moral assessment, thus causing prominence [53].

The commercial channel’s monologue titles prominently feature references to the coronavirus pandemic, such as "The Second Wave," "Once again: Closure," "On the Way to Vaccination," "The End of the Closure Era," "Red State," "The Opening of the Program: Corona," "Back to Life," and "Curfew." Notably, most of these titles have negative connotations, aligning with the general tone of the monologues, which often criticize and use negative language. The use of COVID-19-related terminology in the titles is consistent with the use of similar phrases and visual symbols throughout the program. In contrast, monologues on the public channel are brief and lack titles altogether.

On the commercial channel, there is much use of graphs or large titles of numbers and percentages to illustrate numerical data. On June 26, 2020, these elements were used eight times, and on July 17, 2020, they were used nine times in the same monologue. All monologues feature graphs or large titles of numbers and percentages. Throughout the monologues, there is a lot of text related to numbers, percentages, various comparisons, and predictions for the future. Graphs and titles help illustrate complex information in a flowing and simple way for viewers, which is why they are used so frequently. On July 17, 2020, these elements were used nine times. When comparisons are made between the State of Israel and the rest of the world or between different strata and sectors within the country, the titles and graphs become extremely useful for illustrating the differences and comparisons within them.

In contrast, the public channel does not use graphic representations or large titles to illustrate numbers and percentages. Only one such element is used in a monologue presented on July 24, 2020.

In summary, visuals play a crucial role in conveying a message, and the use of visuals, including pictures and videos, as well as titles and graphs, can differ significantly between commercial and public channels, impacting the overall effectiveness of the message.

### "Mental distress": Use of negative framing that creates a presentation of panic

It is evident from all the samples in both channels that there is a greater use of negative words compared to positive ones. This is in line with the idea that the framing of news directly influences public opinion [3] and that negative information has a stronger impact than positive information, as it is more memorable, prominent, and persuasive than professional arguments [6]. In commercial channels, negative words are expressed through the choice of language as well as through negative content. For instance, on June 19, 2020, phrases such as "a record in the number of infections," "the numbers have indeed increased," "patients in a serious medical condition," "the coronavirus is not going anywhere," "demagogue," "a disease we do not truly understand," and "(the politicians) broke a promise" were used to provide a summary of the first six months of the coronavirus period. On July 3, 2020, attention was drawn to the economic challenges faced during this period, with negative expressions such as "disturbing news," "record in the number of infections," "the number of deaths thus far," "one of five Israelis without a job," and "groaning under the economic corona." On January 22, 2021, the general trend was that of war with "difficult and protracted battles," "the vaccine hits, the mutations return fire," "the number of infected is soaring," "we will feel the results for years to come," "a series of omissions," and "conflicting ministers." Furthermore, negative expressions such as "damage for years," "the government was wrong," "loneliness," "mental distress," and "economic crisis" appeared on February 19, 2023. Additionally, criticism of the government and its conduct is typically presented in negative terms.

Moreover, it is apparent that the language used in commercial channels is often emotionally charged with words and phrases that evoke strong feelings such as "peak," "distress," and "rebellion." They even evoke the repeated traumas of Israel as a region where wars frequently occur. Positive expressions are sparse and are limited to phrases such as "we are starting to get out of the corona epidemic," "there are reliefs," or "the vaccine is working." Negative words and content are reinforced by the use of "warning colors" in red and orange shades on June 26, 2020, July 31, 2020, January 22, 2021, and January 26, 2021. Additionally, the music that accompanies most of the monologues is repetitive and conveys a sense of pressure, tension, and war.

Negative expressions are further enhanced by images, videos, bold headlines, and numerical graphs that reinforce the tone of the message. Frequently, when morbidity or mortality data are presented, numerical graphs are used to illustrate information. The headlines themselves are anxiety-provoking, such as "The Second Wave," "Again: Lockdown," "Red Country," "Opening of the Program: Corona," and "Curfew." Furthermore, when the host presents negative content, they often use a sarcastic, cynical, or indifferent facial expression and tone of voice, suggesting a sense of resignation that "there is nothing to be done, this is the way it is, and so it will be."

On the public channel, there is a notable emphasis on the negative aspects of the epidemic, particularly in terms of politics and healthcare. For instance, during the monologue on February 26, 2021, eight negative phrases were utilized, such as "We will all pay the bill," "He decides on his own when and where the money will go," and "There is no parliamentary oversight." Conversely, only two positive phrases were used, "Helps" and "Good that the vaccines were purchased." On other dates, it can be observed that there is a complete absence of positive words, in stark contrast to a plethora of negative expressions. For example, on September 4, 2020, six expressions with negative connotations of helplessness were used, including "At a loss," "Looking for a scapegoat," "No initiative," and "Lost control," with no positive words being employed in the entire monologue. A similar trend can be seen on July 3, 2020, when eight negative expressions were used, such as "The creaks in the government have become jarring," "They are exchanging blows," and again no positive words were used.

### The voices of the people

The present study reveals that both the commercial and public news channels demonstrated a lack of attention to the voice of the people, whether positive or negative, in both channels. While such a trend aligns with the profit-driven motives of commercial channels, it is not consistent with the principles that ought to guide public news channels. Specifically, public news channels are expected to prioritize a balanced and multifaceted perspective, characterized by the provision of background and contextual information and the promotion of diversity of opinions in the interest of public value and service [20].

The present study has revealed that the public channel failed to incorporate people’s voices into its news coverage to a significant extent.

On the other hand, the commercial channel exhibited a similar tendency, with the exception of specific dates such as February 19, 2021, where five positive mentions were made, and July 31, 2020, where only two negative mentions were made. Notably, in Channel 11, the public channel, the people’s voices were almost completely absent from the monologues, and on rare occasions where they were featured, they were presented in a negative light, serving as a platform for criticism.

The present analysis suggests that monologue speakers hold the power to shape the discourse around the epidemic in both commercial and public news channels. It appears that they tend to express their opinions and ideas without seeking input from the public.

As previously noted, the voices of the public are almost entirely absent from the monologues on the public channel, and when they are heard, it is usually in a negative context conveying criticism that aligns with the monologue’s intended message. For instance, on September 18, 2020, there was social criticism of the government, and a similar critique was voiced from an economic standpoint on July 31, 2020.

This lack of representation of the people’s voice may be a cause for concern, particularly in the case of the public channel, which is expected to promote diversity of opinions and universal service. It is possible that this trend reflects a broader issue in contemporary media where the media’s agenda is primarily shaped by elites rather than reflecting the interests and concerns of the public.

On February 19, 2021, as Israel prepared to turn green and ease restrictions, the background of the monologue featured several well-known artists, athletes, and actors, symbolizing the return of these professionals to their respective fields. The imagery portrayed positive voices from the industry in favor of the lifting of restrictions. However, these individuals were not ordinary citizens. In contrast, on July 31, 2020, the monologue background featured two protesters who were attacked during demonstrations against the state. This imagery emphasized the negative voices of people on the ground, highlighting chaos, disorder, and lack of control.

These few references from the field appear to reinforce the overarching narrative presented by the monologues: criticism of the government’s management of the crisis and a call for the economy to reopen and restrictions to ease. It appears that monologue speakers hold the baton, and they prefer to voice their opinions and dictate them without soliciting feedback from the public.

In line with the findings of the current study, it appears that the voices of the people are largely absent from the monologues on the public channel. When these voices are present, they tend to be expressed in a negative manner, conveying criticism that reinforces the dominant themes of the monologues. Specifically, on September 18, 2020, social criticism of the government was voiced, and a similar criticism from an economic perspective was heard on July 31, 2020. Overall, the public channel’s failure to provide a platform for a diversity of opinions from the public appears to be in contradiction with its mandate to provide universal service, public value, quality, and diversity of perspectives [20].

### "They do not control the event": Criticism of the government

The prevailing trend in both channels is criticism toward the government. It is worth noting that television news generally tends to have a negative tone and often highlights criticism and common problems [20]. On July 17, 2020, the government’s handling of the pandemic was criticized in both channels with phrases such as "They are not in control of the event" and "The situation will get worse". Similar criticism was expressed on September 4, 2020, with statements such as "Failed management" and "Failing grade". These criticisms reflect the general negative tone.

Despite the expectation that the public channel would be more moderate in its views and show support for the government and its actions during the crisis, the reality is that there has been a clear trend of criticism toward the government in both channels. This is contrary to the idea that public media should provide a platform for a diversity of opinions, as enshrined in the concept of freedom of information in a democracy [1].

In the commercial channel, there is a consistent trend of criticism toward the government, with very little support for the government being expressed. For example, on July 24, 2020 and September 18, 2020, the government was criticized six times, and on July 31, 2020 and September 4, 2020, such criticisms appeared four times. Negative words such as "failed management", "(the government) is at a loss", "(the government) is led and does not initiate", "the crisis of trust (between the people and the government) only deepens", "anarchy", "the government is wrong! ", "there is no parliamentary oversight", and others are frequently used in conjunction with these criticisms. The feeling of the government’s ineptitude in the crisis is further reinforced when comparisons are made between Israel and other countries in the world, as was done on July 3, 2020, July 17, 2020, and September 4, 2020. Additionally, comparisons made within the State of Israel between different sectors also emphasize the government’s failure to manage the crisis properly. Such comparisons can be found in half of the monologues.

In addition, the use of visual aids such as pictures and graphs reinforces the negative criticism of the government. When the monologues criticize the government, pictures of various members of the Knesset are often attached, sometimes in a normal representation and sometimes in a satirical way. The use of graphs and large titles with numbers and percentages also serves to emphasize the difficult and dangerous situation of the state, indirectly alluding to the government’s flawed actions that led to the results shown. Comparisons between Israel and other countries, as well as comparisons made within the various sectors of the country, further strengthen the negative criticism of the government and emphasize its inadequate conduct according to the mindset presented in the monologues.

Throughout the monologues on the public channel, a recurring theme of government criticism is evident. Examples of such criticism include statements made on July 3, 2020, that "the squeaks in the government have become shrill," on February 19, 2021, that "there is no transparency, the Knesset is isolated," and on January 22, 2021, that this is a "failed policy" and "problematic enforcement." Positive words toward the government are infrequently expressed. The majority of criticism centers on the government’s handling of the pandemic, such as "farce," "slight embarrassment," "clumsiness," "big headlines and then the implementation test," and "promising but not promising to fulfill," as was stated on July 24, 2020. Criticism of the government appears to have intensified as progress is made in managing the epidemic, with trust in the government decreasing. Although positive statements about the government were made upon the arrival of vaccines, such as "Israel purchased vaccines and it is a good thing," "The country will be painted green," and "Starting to come out of the epidemic," criticism is still present in such monologues.

## Discussion and conclusions

The outbreak of the global COVID-19 epidemic caused widespread panic among the public and resulted in extensive media coverage. With its unknown consequences, the virus-containment measures included isolation, lockdowns, and extended stay-at-home orders. The media thus became the primary mediator between Israeli citizens and the outside world. In addition to objective coverage, Channel 11 and Channel 12, the public and commercial news channels, respectively, began presenting opening monologues by their main hosts Yaron Dekal and Danny Kushmaro. The monologues focused on the reality of the new and unfamiliar COVID-19 epidemic, expressing criticism of decision-making and public leaders’ behavior.

This study examined the framing differences between the opening monologues of the public and commercial channels based on framing theory, which posits that news framing directly affects public opinion. Commercial news is typically sensational and contains more soft news topics such as crime (like a good movie script), while public news promotes a more balanced view and is expected to describe news in a more positive way [20]. Contrary to the literature [1], this study found a similarity in the negative emphasis of the COVID-19 epidemic across both public and commercial channels. This may be attributed to competition with commercial channels, as public channels strive for ratings and sometimes aim for programs that highlight their "scarcity of resources" [16]. Additionally, the study found that commercial channels use a wide range of negative words and express criticism of the government, while positive aspects are rarely heard. This contradicts the literature, which suggests that the media typically supports the government during a crisis [28].

The media has a great influence during times of crisis, as it can cause a calming response and prevent panic, thus avoiding escalation in a crisis [6]. In commercial channels, there is much emphasis on negativity and negative outcomes, while public news has a more positive tone [20]. Accordingly, this study found that commercial channels conveyed a message of panic and chaos regarding the COVID-19 crisis, while public channels attempted to motivate action by the government and the public to overcome the crisis [54].

Visual information is better assimilated than verbal information, and television news often relies on visual images, which produce a stronger emotional effect than still images [5]. The study found that commercial channels used a significant number of visuals, including still images and videos, to make it easier for viewers to accept the information presented. In contrast, the public channel’s monologues were accompanied by fewer pictures, videos, and graphs, and at the beginning of the epidemic coverage, there were almost no pictures at all.

Commercial channels focus on maximizing profits and aim to generate significant consumption by concentrating on large and sensational headlines [17]. On the other hand, public news should include content elements, political knowledge, interest, democratic values, and public capital, as it is free from commercial pressures and appointed by the state. This study found that the commercial channel made extensive use of headlines, visuals, and videos, while the public channel concentrated on the conveyed information without using such graphic elements. The dramatic presentations and videos may make watching the commercial channel more attractive to viewers, but they may also cause them to forget the depth of the information presented and hinder objective thinking.

The study’s novelty lies in its coverage of a crisis of a magnitude that arguably modern media has not experienced. During the crisis period, both commercial and public media channels appeared to have behaved similarly in shaping public opinion. They were critical of the government’s conduct, utilized negative framing extensively, and did not provide a platform for popular voices. The crisis seems to have diminished the previously evident distinctions between the commercial and public channels, indicating that the crisis eroded the unique elements that differentiated them. This outcome can be attributed to the unprecedented nature of the coronavirus crisis, which the modern world has arguably not experienced before. The coverage and framing of both commercial and public channels were unified due to the severity and novelty of the crisis. COVID-19 was not an exclusive problem for Israel but rather a global issue, so the media utilized a broad range of sources beyond the national domain. Consequently, the media’s coverage was influenced by global trends, leading to the blurring of the differences between the characters of the various channels.

Moreover, it may be posited that the unique nature of the coronavirus crisis generated a higher level of fear and panic compared to previous crises, contributing to increased criticism of the government’s policies and the widespread use of negative framing, as observed in both channels.

## Conclusions

In conclusion, the present study reveals a significant prevalence of negative terminology and sharp criticism of the government, as well as prominent coronavirus concepts in traditional media coverage of the COVID-19 pandemic. To attract consumer attention, the framing of the crisis in traditional media frequently incorporates negative words such as "Disturbing news," "Record in the number of infected," "One in five Israelis without a job," and "Groaning under the economic corona," which can increase media consumer anxiety [24]. The media’s choice of spokespersons and emphasis on risks and uncertainty can also contribute to the level of panic in society [24].

Previous research indicates that during a "surprise crisis," the media often mobilizes and voices the dominant voice out of helplessness and lack of knowledge [25]. However, the present study shows a prevalence of criticism toward the government during the COVID-19 epidemic, although it is a "surprise crisis", possibly due to the media’s inability to be the government’s mouthpiece in the current era, where social media serves as a key channel for authentic communication [28].

In times of health crises, social media serves as a vital means of communication that enables the identification of authentic voices, which are deemed more trustworthy than those that may be influenced by parties with vested interests [23, 55].

Therefore, the media’s credibility can be undermined if it blindly supports the government, as consumers can cross-check and verify information through social networks [28].

The media’s partial criticism of the government, despite supporting its policies of lockdowns and vaccinations, may be due to the perception that the COVID-19 pandemic did not pose a significant threat to humanity, and the situation was expected to be under control. Additionally, governments are expected to be prepared for epidemics, which most governments were not. The competition between public and commercial channels for ratings has resulted in public channels also targeting programs that emphasize the "scarcity of resources," ultimately competing with commercial channels. This raises the question of whether public broadcasting fulfills its role toward society if its coverage is almost the same as that of commercial channels. This highlights the need for public broadcasters to uphold their mandate of providing diverse perspectives, promoting democratic values, and offering content that goes beyond sensationalism.

The present study supports malaise media theory [31, 34], which suggests that news coverage places greater emphasis on intrigues, personal stories, scandals, and emotional discourse than on "pure" politics to attract media consumers and increase exposure and ratings [33]. Previous research indicates that media coverage of the crisis produces anxiety among consumers [35]. The present study reveals how negative terminology and powerful concepts activate emotions and contribute to media-induced anxiety. It is crucial for the media to practice responsible and balanced reporting, considering the potential consequences of their framing choices. Accurate and balanced reporting can prevent the escalation of fear, ensure accurate public understanding, and promote public trust, ultimately leading to informed decision-making and better mental well-being for the population.

## Limitations

The research conducted in this study is limited to analyzing the monologues that open the newscasts, and, as such, there is a possibility that the coverage of the rest of the programs may differ in some way. However, it is believed that these monologues provide a glimpse into the content of the program and its key themes. Future studies may be able to provide a more comprehensive understanding of the nature of the coverage of the commercial and public channels during the coronavirus crisis by analyzing the content of the main programs as well. Such studies would be valuable in providing a more nuanced understanding of the media’s role in shaping public perception and attitudes toward the crisis and may have important implications for public health policy and communication.
